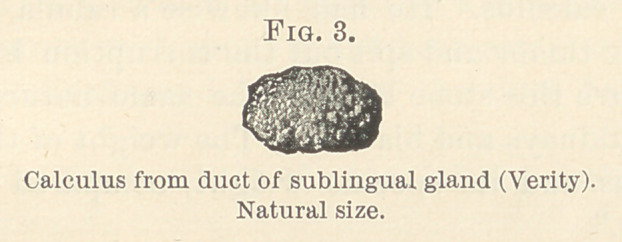# Salivary Calculi

**Published:** 1896-10

**Authors:** Gustav Fütterer


### International Dental Journal.

Vol. XVII. October, 1896. No. 10.

### Original Communications.[1]

### SALIVARY CALCULI.[2]

BY GUSTAV FÜTTERER, M.D.[3]

A few months ago I extracted two calculi from a submaxillary gland, and, as such calculi are very rare occurrences, I thought it well to report the case; Professor Christian Fenger then favored me by putting at my disposal a calculus which he had removed from Wharton's duct, and Dr. W. P. Verity kindly gave me another, which he had taken away either from a sublingual gland or from one of its ducts.

Case I.: *Submaxillary Calculi.*—Mr. M., thirty-four years of age, about four years ago, while eating, noticed the appearance of a swelling under the jaw, on the left side. A moment's pressure of his finger on the swelling caused it to disappear. This occurred several times. Three years ago this spring he noticed a similar

[1] The editor and publishers are not responsible for the views of authors of papers published in this department, nor for any claim to novelty, or otherwise, that may be made by them. No papers will be received for this department that have appeared in any other journal published in the country.

[2] This article, by Dr. Fütterer, on "Salivary Calculi," is reprinted from *Medicine*, a new medical journal, by special request. Our rule not to republish papers under the original heading is laid aside, in this instance, on account of its special value and, further, that dental readers will not, as a rule, have the opportunity otherwise of referring to it. The very extended bibliography will be of great value to future writers upon this subject.—Ed.

[3] Professor of Physical Diagnosis, Chicago Policlinic; Attending Physician, Cook County Hospital; Attending Physician, St. Elizabeth Hospital; Consulting Physician, Deaconess Hospital.

swelling, which, however, became enlarged and painful; pressing had no effect. A physician was called, who prescribed poultices and leeches, and after a week the swelling gradually disappeared. After about six months the same trouble came again, but this time it did not yield to treatment as readily as before. After about another six months it again made its appearance and was more stubborn than ever. Altogether the patient had suffered from such attacks four or five times when he called upon me.

I found him a tall, slender, somewhat anæmic man, with a "weak" stomach and an enlarged gall-bladder,—he had suffered from several attacks of gall-stone colic; he complained of pains in the left submaxillary region and some difficulty in swallowing. The left submaxillary region was swollen; there was a swelling on the floor of the mouth, along Wharton's duct; and on pressure of the submaxillary gland, pus of a greenish color escaped from the orifice of Wharton's duct on the left side. A calculus could not be felt, but the frequent attacks on the same side, in the course of years, together with the fact that the patient had never suffered from tonsillitis, pharyngitis, or stomatitis, suggested the idea that a calculus might be the cause of the trouble.

I then called in Dr. John S. Marshall for surgical aid. Dr. Marshall passed a small silver probe into the duct and discerned, at a depth of seven centimetres from the cutting edge of the inferior central incisor tooth, a hard body, which led to the belief that a calculus was present in the gland. He then cut open the upper third of the duct, and introduced an especially prepared slippery-elm tent, intending to remove the tent after a lapse of twenty-eight hours and look for a calculus.

In the course of the next night the swelling and the pains increased considerably, involving the left side of the face and neck, and the whole tongue, compelling me to remove the tent after it had been in position for twenty-four hours. The removal of the tent was followed by a gush of about an ounce and a half of saliva and greenish pus. On passing a probe, we felt a calculus deep down in the gland. Dr. Marshall then passed a grooved director down to the calculus, and with a curved bistoury laid open the duct throughout its entire length. The incision measured about two centimetres at the surface of the floor of the mouth, but probably not more than one centimetre at the level of the calculus. This, however, did not enable him to take hold of the calculus and extract it. My own attempts in the same direction also failed, and we therefore packed the incision to further dilate the duct and make another attempt the following day. The next morning, after removing the packing and washing the gland thoroughly with a five-per-cent. solution of carbolic acid and then with peroxide of hydrogen, through a rubber catheter, I succeeded in removing a small faceted calculus of a yellowish color, weighing one grain. On the same evening I removed another calculus, also of a yellowish color, weighing twenty-four grains. A

Fig. 1.

Calculus from submaxillary gland (Fütterer).

Natural size.

thorough probing of the cavity convinced me that no more calculi were present, but for greater certainty I introduced a urethral electroscope, and, as the bed of the concretions was in the upper posterior portion of the gland, I had no difficulty in submitting the parts to ocular inspection. If the thought of using the electroscope had suggested itself to me sooner, I could have easily ascertained the size, shape, and position of the calculi, and this would have facilitated their extraction materially.

During the following two weeks the cavity was cleaned twice a day, and after each cleaning the duct was loosely packed with gauze to prevent foreign bodies from entering. Later on, when the duct grew smaller, a silver tube was introduced and allowed to remain. Twice this tube was changed for a smaller one, and now the patient wears one that has about the diameter of a normal duct. All those tubes I fastened to the incisor teeth with silver wire.

*Chemical Examination.*—Dr. J. A. Wesener, who made the chemical examination, reports as follows: "Specific gravity, 1584. It is composed of calcium, sodium, and potassium acid phosphate, with a trace of xanthine and iron. Tests for ptyalin, potassium sulphocyanide, fats, fatty acids, carbon dioxide, magnesium, and inorganic acids, negative."

The microscopical examination (Fütterer) gave no particular results.

Case II.: *Calculus from Wharton's Duct.*—The following history was kindly given me by Professor Christian Fenger, who observed the case:

Mrs. X., fifty years of age, on partaking of sour food, would occasionally notice a little lump arising in the right submaxillary region, accompanied with some pain. In half an hour the lump would disappear, and would not return for two or three months. So it ran along for years. The patient never had any sore throat or stomatitis until about two months before operation, which was performed on the 21st of January, 1895. The lump, however, had been continuously present for some time, the submaxillary swelling tender, and the throat and right half of the floor of the mouth sore. Swallowing was painful all the time, and she had been gargling with listerine since the latter part of November to cure what she considered to be a sore throat. Sometimes when she sat down to a meal, the lump would assert itself very suddenly, with a kind of dull pain, which would continue for fifteen or twenty minutes, sometimes an hour, or even on rare occasions an hour and a half. Then the swelling would disappear spontaneously. She never noticed that any fluid came into her mouth, or that she had to spit; the swelling simply disappeared. When the swelling was present there would be a sensation of pain and distress in swallowing.

Under anæsthesia the duct was opened for three-quarters of an inch, and with forceps a stone was removed from behind and below the posterior border of the mylo-hyoid muscle. The cavity was about two centimetres long and one centimetre wide. The duct was found dilated. The cut portion was united by sutures, and healed almost entirely by first intention. After healing, a probe was passed freely from an opening close to the papilla to the posterior part of the duct, where an opening into the mouth remained, through which saliva or clear mucoid fluid could be squeezed up by pressure on the gland. Ten days after the first operation, Professor Fenger, without an anæsthetic, closed this opening by sutures; after three weeks it was closed permanently.

Fig. 2.

Calculus from Wharton's duct (Fenger).

Natural size.

An examination made April 18, 1896, showed that the patient had remained perfectly well; sublingual and submaxillary glands normal, also Wharton's duct; no opening at posterior border of mylo-hyoid muscle; a probe could be passed through a small opening two millimetres outside of the papilla for a distance of two to three centimetres; duct not dilated.

*Chemical Examination.*—Specific gravity, 2306. Stone composed of calcium, sodium, and potassium acid phosphate, with a trace of iron and uric acid.

CASE III.: *Calculus from a Duct of the Sublingual Gland.*—Dr. W. P. Verity gives me the particulars of a remarkable case. His patient was a woman aged forty. At the age of twelve she began to feel a swelling at the left side of the floor of the mouth, which would come and go, and which interfered with mastication, but not with swallowing; twenty-eight years later the swelling had increased so that it lifted up the tongue and pressed it over to the right side. Interference with mastication increased, but there was no difficulty in swallowing. A calculus could be felt about one and a half inches from the caruncula salivalis. Dr. Verity cut into the mass; pus was evacuated, and with it a calculus.

FIG. 3.

Calculus from duct of sublingual gland (Verity).

Natural size.

The calculus was of cylindrical shape, the ends rounded off, flattened on one side of its longest diameter so that a cross-section would show a plano-convex shape; surface warty, color grayish-white, and consistency hard.

Avenzoar, who lived in the thirteenth and fourteenth centuries, seems to have been the first to recognize the presence of salivary calculi "under the tongue." The earliest cases of which I have read the account in the original were reported by Lister[1] in 1672, and by Bonavert[1] in 1698.

Lister reports: "After a severe cold a patient noticed a hard lump in his mouth. This was due to a calculus, and about eight years passed between its breeding and its being taken away." As to its growth and the inconveniences thence ensuing, he further says, "Upon all fresh cold-taking, he suffered much pain in that part, and yet, that cold being once over, the part was no more painful than the rest of the mouth." Lastly, as to the particulars remarkable at the time of its being taken away, he relates: "It began its work with a sudden vertigo, which lasted from spring till August, in which month, without any previous cause, save riding, the place where it was lodged suddenly swelled and emitted purulent matter

[1] Philosophical Transactions, London.

at the aperture of the Whartonian duct. Then it suddenly stopped its running, and swelled with great inflammation, and very great danger of choking, causing great pain when endeavoring to swallow anything liquid. Incision; removal of a whitish calculus, which weighed seven grains."

Bonavert relates his case as follows: "Thomas Wood, of Wrotham, was so troubled with a quinsy that he could hardly swallow any liquid. I found the tumor tend to suppuration inwardly, about the root of the tongue on the right side, though it was almost as large as an egg outwardly, but without any sign of suppuration there. I ordered him maturating gargles, and the next day sent my man and bid him advise him to endeavor to break it with his finger, which the man effected and brought out of his mouth near the quantity of a quarter of a pint of matter, and with it, at last, the calculus. He had likewise a ranula, and before he had broken the tumor and spit out the corruption he could hardly speak. I believe this stone to be of the same nature as those generated in the kidneys and bladder. The weight of this stone in air is seven grains, and its specific weight, compared with water, is near $1\frac{91}{103}$ to 1."

Before the year 1800 the following writings dealt with our subject:

LISTER: Philosophical Transactions, London, 1672.

BONAVERT: Philosophical Transactions, London, 1698.

LISTER, M.: A stone cut out from under the tongue. Philosophical Transactions, London, 1700, iii. p. 155.

SCHERER, C. A.: De Calculis ex Ductu Salivali Excretis. Argentorati, 1737.

HARTMANN, P. L.: Calculum Sublingua Excretum Describit. Helmstadii, 1762.

WÜRGER, F.: Bemerkung von einem Speichelsteine. Kopenhagen, 1778.

TITIUS, S. C.: De Calculo Salivali, Sponte Excreto Observatio, 1794.

Immisch, in 1861, opposed the opinions of English physicians, that there was a practical relation between the origin of salivary calculi and gout. His own opinion was as follows: "Inflammations of the salivary ducts are frequent occurrences, and if such inflammations become chronic they may cause small elevations and indentations which will narrow the lumen of the duct, thereby causing a retention of saliva. Saliva will be retained in folds and pockets, and crusts form, to which mucus and pus are added. This crust leaves open the centre portion of the duct, and so the canal is formed which has been found in salivary calculi. In the interior of the gland the calculus is formed in the same manner, but here the calculus has no central canal."

Mareau accepts three causes: first, foreign bodies which have accidentally entered the duct; second, tartar (hypothesis of Richet); third, inflammatory strictures.

Let us see what material literature gives us for reasoning in this direction.

De Closmadeux (1855) says that in two cases foreign bodies had been found.

S. Michel (1867) describes the formation of a calculus in a duct of the sublingual gland, after this duct had been pierced through by a fish-bone, the latter forming the nucleus of the calculus.

J. W. Hulke (1872) found a dark central speck, which proved to be a fragment of wood.

Rochs (1894) saw shot as a nucleus, in a musician who used to clean his instrument with shot; it had been aspirated, pressed into the caruncula salivalis, and by way of the duct had entered the gland.

Rayer found a foreign body the size of a gooseberry-seed as a nucleus.

Roberts (1869) saw a man fifty-four years of age who, twenty years before, had eaten some mustard and got a small mustard-seed under the tongue, which caused him violent pain for some days. The pain finally subsided, but the patient felt a small lump there ever after. This lump under the tongue would swell and pain him whenever he took cold, but the trouble would disappear with the cold. Dr. Roberts removed a calculus, and in its centre found a cavity "very much the size and shape of a mustard-seed," but he found no mustard-seed.

In the foregoing six cases foreign bodies were ascertained beyond a doubt as the nuclei of the calculi, while in one case it seems certain that a mustard-seed had been there but had disappeared.

Kochling (1835), who published a case of calculus, thought it necessary to mention that the teeth of his patient were covered with thick layers of tartar, and Mareau and Richet also call attention to tartar as a probable cause.

Wyatt Pratt (1871) had a patient who had once shown symptoms of tuberculosis of the lungs, and had coughed up from the bronchial tubes some calcareous concretions, while later came a number of calculi from Wharton's duct.

Gross foreign bodies which enter the mouth with the food, or in a more accidental manner, can become the cause and the nuclei of salivary calculi. This has been found true in a comparatively small number of cases. Extraneous materials present in the oral cavity, breaking loose and entering the ducts, can cause the same effects. I refer to fragments of decayed teeth, and especially tartar. The fact that chemical analysis revealed in my own case xanthine, a derivative of uric acid, and in Professor Fenger's case uric acid, points to tartar as a cause, as tartar sometimes contains uric acid. Dr. J. A. Wesener, who has analyzed the tartar of one hundred teeth, found uric acid in eight of his specimens.[1] Small pieces of tartar often break down and can very easily enter the ducts, especially Wharton's duct, and here we find the most calculi, which are very rare occurrences indeed in Steno's duct. Gravitation will bring the pieces down to the bottom of the mouth rather than up to Steno's duct; they will also remain there longer, and occasion to enter will offer itself more readily. The lower incisors are places of predilection for the formation of layers of tartar, which here project as plates over the margin of the gums and easily break down.

Bacteria, especially leptothrix buccalis, may give rise to the formation of a calculus. Bacteria have so far, according to my knowledge, not been found in salivary calculi, and I have not found them in my cases, but that is of little importance if we remember that a mustard-seed could have disappeared, and that tubercle bacilli seemingly disappear in old fibrous and calcified tubercles, and if we further consider the length of time needed for the formation of a stone.

A certain disposition for calcareous depositions is indicated by the case of calculus of Wharton's duct of Wyatt Pratt, in which calcareous concretions were coughed up, and also by my own case, in which the patient had an enlarged gall-bladder and had suffered from occasional attacks of gall-stone colic.

I have reviewed forty-five cases of calculus in the duct of the submaxillary gland, nine cases from the submaxillary gland, four cases of calculi which seem to have occurred in the sublingual gland, and four in its ducts. So the calculi are chiefly found in Wharton's duct, while they are very rare in the submaxillary gland and in the sublingual gland and its ducts. I may add that only a few cases of calculi have been found and reported in the parotis and Steno's duct in man, while a great many have been reported in animals. If to our number of cases, sixty-seven, we add the ninety-

---

[1] International Dental Journal, April, 1896.

three reports which we did not have at our disposal, the number of salivary calculi reported would sum up one hundred and sixty; and bearing in mind that these are the cases reported from the thirteenth century up to date, we must come to the conclusion that salivary calculus in human beings is a rare occurrence, even if we grant that there may have been cases in which the nature of the trouble was not recognized, and other cases which have never been published. Concretions were found in only three cases by Virchow, Closmadeux, and Malenfant, and they all occurred in Wharton's duct.

Men are affected about ten times as frequently as women.

The earliest age at which the symptoms have appeared was twelve years, and twice we find the age of seventy reported as the time of operation and relief, but from the twentieth to the fortieth year is the preferred time of life.

One calculus was found in fifty-five cases, ten calculi in one case, and a great many in one case.

*The symptoms of calculi of the submaxillary gland and its duct* may be classified as follows:

1. *Symptoms of the Initial Stage.*—Only in one case was this stage well marked by severe pains, caused by the entering of a mustard-seed (Roberts), which then caused the formation of a calculus.

2. *Symptoms of the Stage of Formation of a Stone and of its Growth.*—This stage may be passed through without any noticeable symptoms arising. Bruce reports a case in which a calculus existed for fourteen years without causing much inconvenience. Most patients on eating, especially if the diet be particularly tempting (Elston), will suddenly notice a swelling in the submaxillary region, which according to its degree may be more or less painful. On resting the mouth and pressing on the swelling the latter will disappear. Such swellings will also come and go with colds (Lister, Roberts). They appear in the submaxillary region, are of a hard consistency, and are also to be seen and felt at the floor of the mouth between jaw and tongue, pressing the latter upward and somewhat to the other side. In one of Hulke's cases the swelling seemed to be so firmly grown onto the hyoid bone that it was taken to be a fibroid. If the calculus is lodged in Wharton's duct, it can often be felt by the patient or the physician. Alston's patient complained of being unable to eat, of feeling a weight, and of having a rock in her mouth, while a patient of Freudenberg had noticed a calculus which projected and could be seen close behind the right caruncula salivalis. The voice also may be affected. Clark, in speaking of a patient, says, "His voice, which had been harsh and coarse, after removal of the stone became flexible and resonant." Severe attacks of toothache, caused by the presence of a calculus, have also been observed at this stage, and Lister reports vertigo lasting from spring until August. Elston says, "The sympathy existing between the nerves of smell and taste was in my case most beautifully illustrated, for, according to the patient's account, he could never pass a savory smell without feeling a sudden enlargement of the submaxillary gland and pain, and he said he had dined but a few days previous to my seeing him on a meal which always used to make his mouth water, but which, in this instance, in consequence of the outlet of the duct being completely closed, had produced so violent a distention of the gland as to at once set up such a degree of active inflammation as shortly after led to the discovery of the nature of the disease."

3. *Symptoms of the Stage of Suppuration.*—Suppuration prepares for the expulsion of the stone, which in many cases is brought about by way of the duct or by way of a fistula, of which I find three cases reported, in one of which Nélaton extracted a calculus through a fistula. Such a suppuration may come suddenly, causing considerable swelling of the gland and the surrounding parts, great difficulty in swallowing and in mastication, impaired speech (Bonavert), facial pains (Rouyer), attacks of suffocation (Lister, Jessup), and in Oliver's case the opening of the mouth was prevented by the painful swelling. In short, this stage brings quite a variety of symptoms and a great deal of suffering to the patient, who soon seeks relief.

The stage of suppuration may, however, come and go several times, or it may become extremely chronic, as in Terrier's case, where chronic swelling and discharge of pus lasted for a long time.

*Symptoms of Calculi in the Sublingual Gland and its Ducts.*—Immisch (1891) considered the formation of calculi in the sublingual ducts improbable. Michel (1867) reported a case, which I have already mentioned, in which a fish-bone had pierced a sublingual duct, and then a stone had formed. And I think all doubts are removed by Dr. Verity's case, in which there was no difficulty in swallowing but great difficulty in mastication, much swelling in the mouth but very little to be seen in the submaxillary region, and while there may have been some slight compression of Wharton's duct, the stone could not have been lodged there, but must have been in the sublingual gland or one of its ducts; judging from the shape of the stone and from the fact that it could be felt, I should say it was in a duct. As by far the most calculi are found in the ducts, the symptoms, together with the results of palpation and careful probing, will throw light on the case; the probing becomes especially useful if a calculus is located in the gland where it cannot be felt by palpation.

*Modes of Procedure for removing Calculi.*—Bonavert (1698) sent his man to the patient, and bade him tell him to try and break it (the tumor) with his finger, "which the man effected." J. W. Hulke (1872) made his way to an abscess cavity in the submaxillary gland, from the outside, tying the facial artery. All other operators have opened the abscess wherever they found it, or proceeded by way of the duct, cutting it open. Fenger anæsthetized his patient, cut the duct, and sewed it up again, while Marshall cut the duct and then dilated its lower portion with a slippery-elm tent.

In our sixty-seven cases, five single relapses occurred, while in another case three relapses were reported as occurring in the course of twenty years. In one of those cases the calculus is said to have grown within a year, but I would rather believe it had already been present when the other calculus was removed. Calculi had very probably been left in in some of the other cases also, and it seems as if real relapses were very rare, so that we may consider the prognosis to be good if at the time of operation all calculi present are removed. Other bad consequences, such as stenosis, etc., I have not found recorded.

The calculi reported measure up to six centimetres in length and five and one-half centimetres in width, and they have been found to weigh up to eighteen grammes (two hundred and seventy grains).

Their form is more or less cylindrical, oval or round, or more spindle-shaped. The surface has been found smooth, but usually it is somewhat uneven, very finely granulated or warty.

The color is generally a grayish-white or a yellowish-white, but it may also be brownish.

Their consistency is either hard or fragile.

The cut surface is generally lamellated, and Virchow, on examining microscopical cuts, found regular formations of homogeneous lamella and granular portions of yellowish-green color. In my own case the large calculus is only lamellated in its peripheral portions, while the central part shows an irregular configuration. This stone is from the gland itself, while the two others in the original cases reported were from the ducts and lamellated throughout.

The specific gravity differs very much.

Chemical examination always shows the presence of phosphate of lime, and sometimes carbonate of lime. Malenfant made a quantitative analysis, finding—

| | Per cent. |
|---|---|
| Phosphate of lime | 27 |
| Phosphate of magnesium | 1 |
| Basic phosphate of lime | 60 |
| Mucin insoluble in water, alcohol, and muriatic acid | 4 |
| Ptyalin | 2 |
| Loss | 6 |
| | 100 |

According to nationality, I have found reported seventy-two cases from France; thirty-four from Germany; twenty-five from England, Canada, and Australia; twelve from America; four from Italy; and the others from different other countries. France has had by far the most cases, but I am at a loss to even indicate why this is so.

Altogether I have found one hundred and fifty-eight reports dealing with salivary calculi, and I may have overlooked others. I think it well to give all the bibliography which I have been able to gather, as I have not found a complete list of it anywhere.

| | No. of Cases. |
|---|---|
| Before 1800 | 16 |
| 1800–1830 | 11 |
| 1830–1850 | 33 |
| 1850–1860 | 17 |
| 1860–1870 | 27 |
| 1870–1880 | 49 |
| Since 1880 | 5 |
| Total | 158 |

### BIBLIOGRAPHY.

Previous to 1800.—Avenzoar: Thirteenth Century. Lister: Philosoph. Trans., London, 1672. König: Misc. Acad. Nat. Curios., Norimb., 1691. Lister, M.: Philosoph. Trans., London, 1700, iii. p. 155. Scherer, C. C.: De Calculis ex Ductu Salivali Excretis, Argentorati, 1737. Wolffius: Acta. Acad. Nat. Curios., Norimb., 1744, vii. Baster: Acta. Acad. Nat. Curios., Norimb., 1748. Hartmann: Calculum Sublingua Exsectum Describit, Helmstadii, 1762. Leautaud: Jour. de Méd. Chir. Pharm., etc., Paris, 1765. Acrel: Calculus in Ductu Salivali Whartoniano, Heelk. Waarn. Graventh., 1771. Schultzius:

Misc. Acad. Nat. Curios., 1772. Würger: Bemerkung von sinem Speichelsteine, Kopenhagen, 1778 Titius, S. S.: De Calculo Salivali Sponte Excretu Observatio, 1794.

1800–1830.—Seguignal: Jour. Gén. de Méd. Chir. et Pharm., Paris, 1803, xviii. Hirschfeld: J. für die Chir. Geb., etc., Jena, 1806, iv. Gorham: N. E.: Jour. of Med. and Surg., Boston, 1820, ix. John: Deutsches Archiv für die Phys., Halle, 1820, vi. Von Walther: J. de Chir. und Augenh., Berlin, 1825, viii. Weber: De Calculis Salivalis, Berolini, 1825. Burdach: J. die Chir. und Augenh., Berlin, 1825, vii. Velten: Rusch's Mag. für die Ges. Heilk., Berlin, 1825. Basson: Jour. de Chim. Méd., Paris, 1829. Rouzel: Jour. Gén. de Méd. et Pharm., Paris, 1830. Bedor: Jour. hebd. de Méd., Paris, 1830.

1830 to 1850.—Elston: London Lancet, 1835, i. Sympson: Lancet, London, 1835, i. Lohmeyer: Med. Ztschr. und Ver. für Heilk., 1835. Kochlin: Horn's Archiv, 18[illegible]5. Duriens fils: Jour. de Conn. Méd. Chir. Paris, 1837. Maisonneuve: Gaz. Méd. de Paris, 1839, vii. Poggiali: Rev. de Mém. de Méd. de Paris, 1839. Bonfanti: Effem. de Sc. Med., Milano, 1839. Gros: Precis. d. Trans. Soc. Méd. de Boulogne sur Mer, 1839. Rath: Ztschr. für Chir. und Chir. Osterode, 1841, i. Baker: N. Amer. Arch. Med. Surg. Soc., Baltimore, 1835, ii. Marques: J. Soc. de Sc. Med. de Lisb., 1843, xvii. Melion: Oesterr. Med. Woch., Wein, 1844. Van Camp: Ann. Soc de Méd. d'Anvers, 1844, i. Stanski: Arch. Gén. de Méd., Paris, 1846, iii. Robert: Union Méd., Paris, 1847. Bouteiller: Bull. Soc. Anat. de Paris, 1847. Weihe: De Calculis Salivalibus, Gryphiæ, 1847. Bermewitz: Woch. für die Ges. Heilk., Berlin, 1847. Forget: Gaz. Méd. de Paris, 1847, ii. Goddard: Klinik, Utrecht, 1847, ii. La Russa: Esculapio Napol., Napoli, 1847, xxvii. Montgomery: Proc. Path. Soc. Dublin, 1848. Morton: Trans. Path. Soc. London, 1848, ii. Traill: Monthly J. M. Soc., London and Edinburgh, 1850, xi. Poland: Guy's Hosp. Rep., 1851, vii. Bassow: Mém. Soc. de Chir. de Paris, 1851. Jobert: Compt.-Rend. Soc. de Biol., Paris, 1851. Azam; Jour. de Méd. de Bordeaux, 1852. Closmadeux: Rev. Méd. Chir. de Paris, 1855, xviii. Demorey: Des Calculs de la Gland Sous-Maxillaire, Paris, 1856. Demorey: Gaz. des Hôp. de Paris, 1857, xxx. Blin: Mémoir sur les Calculs Salivaires du Canal de Wharton, Saint Quentin, 1858.

1850 to 1860.—Poland: Guy's Hosp. Rep., 1851, vii. Fleury: Bull. Soc. de Chir. de Paris, 1852, ii. Ollivier: Gaz. des Hôp., 1852. Welery: Revue de Thérap. Méd. Chir., Paris, 1853, i. Manillon: Gaz. des Hôp. de Paris, 1854, xxvii. De Closmadeux: Rech. Hist. sur les Calc. des Canaux Salivaires, Paris, 1855. Lange: Tijdschr. d. Vereen te Bevoord, de Geneesk, etc., 1855, ix. Malenfant: Jour. de Connaiss. Méd., 1855. Husband: Jour. de Méd. et de Chir., 1856. Immisch: La Soc. Anat., Août, 1857. Rouyer: Bull Soc. Anat. de Paris, 1857, xxxv. Jobert: Gaz. d'Hôp. de Paris, 1857. Webb: Trans. Path. Soc. London, 1857. Heller: Ztschr. für Wundaerzte und Geb., Stuttgart, 1858, xi. Jackson: Boston Med. and Surg. Jour., 1860. Immisch: De Salolithiasi Morbo. Lipsiæ, 1860. Burnham: Boston Med. and Surg. Jour., 1860.

1860 to 1870.—Immisch: Deutsche Klinik, 1861, Nos. 44–45. Lancelot: Des Calc. Saliv., Paris, 1861. Grant: Brit. Amer. Jour., Montreal, 1862, iii. Blin: Bull. Méd. du Nord, Lille, 1864. Castro: An. r. Acad. de. Cien. Med. de la Habana, 1864. Dorie: Bull. Soc. de Méd. de Poitiers, 1864, No. 30.

Fischer: Allg. Wein. Med. Ztschr., 1864, ix. Shukowsky: Mosk. Med. Ztschr., 1864, No. 48. Marques: Gaz. Méd. de Strasbourg, 1865, xxv. Dourlen: Des Calculs Saliv., Paris, 1865, x. Virchow: Virchow's Archiv, bd. xx. Paris: Bull. Soc. Anat. de Paris, 1866. Bruce: Tr. Path. Soc. London, 1866, xvii. Rochi: Bull. Soc. Méd. de l'Yonne, Auxerre, 1867. Ballard: Trans. Path. Soc. London, 1867. Warren: Surg. Observ., Boston, 1867. Ilken: Nederl. Arch. f. Geneesen Nat., 1867, iii. Michel: Gaz. des Hôp. de Paris, 1867. Drou: Gaz. Méd. de Lyon, 1868. Lelong: Gaz. des Hôp. de Paris, 1868. Balut: Rev. Méd. de Toulouse, 1869. Fazenille: Allg. Wien. Med. Ztschr., 1869, xiv. Flavard: Marseilles Méd., 1869, vi. Bell: Brit. Med. Jour., London, 1869. Roberts: Richmond and Louisville Med. Jour., Louisville, 1869. Firestone: Amer. Jour. Med. Sci., January, 1869. Clark, C. M: Chicago Med. Exam., 1870, i. Scalzi: Giorn. Med. di Roma, 1870, vi.

1870 to 1880.—Paulet: Bull. Soc. de Chir. de Paris, 1870, 155. Maas: Tagebl. d. Ver. d. Naturf. und Aerzte, Rostock, 1871, x. Wyatt Pratt: Lancet, London, 1871. Jessup: Brit. Med. Jour., Feb. 4, 1871. Stevenson: Guy's Hosp. Rep., London, 1872. Ouillon: Med. Times, Philadelphia, 1872, ii. Bourland: Lyon Méd., 1871. Closmadeux: Gaz. hebd. de Méd., Paris, 1872, ix. Vander: Espt. Bull. de l'Académie Méd. de Belgique, 1872, No. 3. Blas: *Ibid.*, 1872. Ikorof: Zapiski Obstr. Vrachg. Kasani, 1873. Umé: Arch. Méd. Belges, Bruxelles, 1873, iv. Hulke: Path. Tr., London, 1873, xxiv. Ritter: Med. Corresp'bl. d. w. Aerzte Ver., Stuttgart, 1873. Forget: Union Méd., Paris, 1874, xvii. Blas: Bordeaux Méd., 1874. Claudet: Rev. de Mém. de Méd., Paris, 1874, xxx. Cusco: France Méd., Paris, 1874, xxi. Terrior: Jour. de Méd., Paris, 1874. Bell: Edinburgh Med. Jour., 1874, p. 824. Gouguenheim: Bull. Soc. Anat. de Paris, 1875, i. Caushois: Union Méd. de la Seine inf., Rouen, 1875, xiv. Paquet: Bull. et Mém. Soc. de Chir. de Paris, 1875, i. Hicquet: Ann. Soc. Méd. Chir. de Liège, 1876. Le Roy: De Langeviniere Annés Méd. Caen, 1876, i. Parphiantovitch: Mosk. Med. Gar., 1876, xix. Pirondi: France Méd., Paris, 1876, xviii. Throwgood: 1876. Mareau: Étude s. l. Calc. Sal. du Canal de Wharton, Thèse, Paris, 1876. Paré: London Lancet, Dec. 15, 1877. Tanksy: Arch. Clin. Surg. N. Y., 1877, ii. Harteloup: Bull. et Mém. Soc. de Chir., etc., Paris, 1877, iii. Feroci: Comment Clin. di Pisa, 1877, i. Freudenberg: Berliner klin. Woch., No. 48, 1877. Rayle: Lancet, London, Dec. 15, 1877. Ankes: Ueber Speichelsteine, Göttingen, 1877. Heurtaux: Bull. Soc. an. de Nantes, 1878. Alston: N. C. Med. Jour., 1878. Gibbs: Brit. Med. Jour., 1878, ii. Tendjanianitz: Des Calc. Sal. et en particulier de leur Diagnostic, Paris, 1878. Bertin: Union Méd., Paris, 1878. Fiori: Gior. di Méd., Roma, 1878, xxvi. Malassar: Bull. Soc. an. de Paris, 1878. Chevallereau: France Méd., Paris, 1878, xxv. Low: Austral. Med. Jour., Melbourne, 1880. Watt: Missouri Dental Jour., St. Louis, 1880. Ganas: De la Lithiase Salivaire et de ses Rapports avec l'Arthritisme, Paris, 1880. Keckzowski: Essai sur les Calculs Sal. du Canal de Wharton, Paris, 1880.

Since 1880.—Strassmann: Berliner klin. Woch., Aug. 15, 1887. Busch: Verhandl. der Deutschen Odont. Ges., bd. i., 1889. Hulke: London Lancet, Jan. 6, 1894. Buchwald: Ueber Speichelsteine, Diss. Griefswald, 1894. Rochs: Deutsche Med. Ztschr., iv, 1894.

## Figures and Tables

**Fig. 1. f1:**
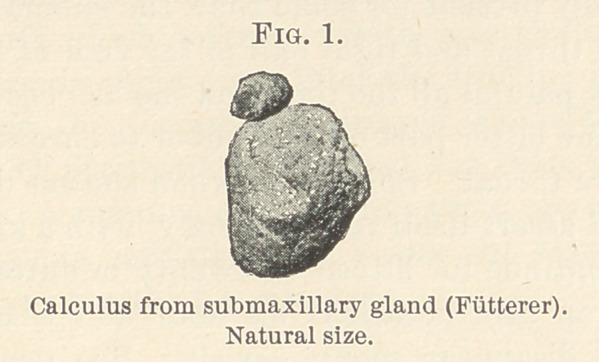


**Fig. 2. f2:**
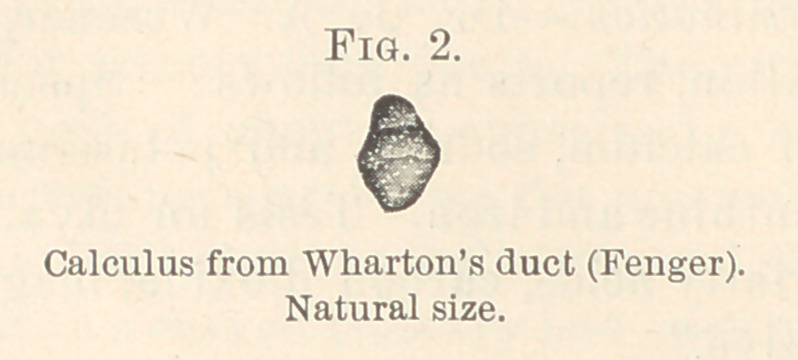


**Fig. 3. f3:**